# Draft Genome Sequence of the Strict Anaerobe *Clostridium homopropionicum* LuHBu1 (DSM 5847)

**DOI:** 10.1128/genomeA.01112-15

**Published:** 2015-10-01

**Authors:** Matthias H. Beck, Anja Poehlein, Frank R. Bengelsdorf, Bettina Schiel-Bengelsdorf, Rolf Daniel, Peter Dürre

**Affiliations:** a Institut für Mikrobiologie und Biotechnologie, Universität Ulm, Ulm, Germany; b Genomic and Applied Microbiology & Göttingen Genomics Laboratory, Georg-August University, Göttingen, Germany

## Abstract

Here, we report the draft genome sequence of *Clostridium homopropionicum* LuHBu1 (DSM 5847^T^), a strictly anaerobic bacterium, which performs propionate fermentation and is capable of growing with 2-, 3-, or 4-hydroxybutyrate as its sole substrate. The genome consists of a single chromosome of 3.65 Mb.

## GENOME ANNOUNCEMENT

The endospore-forming obligately anaerobic organism *Clostridium homopropionicum* is able to ferment various hydroxy- and chlorobutyrates to acetate and butyrate, lactate to acetate and propionate, and fructose to acetate, butyrate, and butanol. The dismutation of 3-hydroxybutyrate to C_2_ and C_4_ compounds obviously plays a central role in its metabolism (1). Despite using the energetically less favorable acrylyl-coenzyme A (CoA) pathway for lactate fermentation, *C. homopropionicum* compensates the lower ATP yield, compared to the methylmalonyl-CoA pathway, by far higher maximal substrate turnover rates (2). *C. homopropionicum* differs from *Clostridium propionicum* (3) in its ability to ferment 2- and 4-hydroxybutyrate (1).

Chromosomal DNA was isolated with the MasterPure complete DNA purification kit (Epicentre, Madison, WI, USA) and used to generate Illumina shotgun sequencing libraries. Sequencing was performed with a MiSeq using MiSeq reagent kit version 3 (600 cycles), as recommended by the manufacturer (Illumina, San Diego, CA, United States), resulting in 2,883,306 paired-end reads (300 bp) that were trimmed using Trimmomatic 0.32 (4). The *de novo* assembly performed with the SPAdes genome assembler software version 3.5.0 (5) resulted in 48 contigs (>500 bp) and an average coverage of 180-fold. The genome of *C. homopropionicum* probably consists of a circular chromosome (3.65 Mb), with an overall G+C content of 31.09%.

Automatic gene prediction was performed with Prokka version 1.9.2 (6). Genes coding for rRNA and tRNA were identified using RNAmmer (7) and tRNAscan (8), respectively.

The genome harbored 10 rRNA genes, 67 tRNA genes, 2,688 protein-coding genes with predicted functions, and 896 genes coding for hypothetical proteins.

With respect to butanol production, the genome analysis revealed that no *sol* operon according to the *Clostridium acetobutylicum* (9) or *Clostridium beijerinckii* type (10) is present. Several copies of coenzyme A transferase genes (*ctfAB*) are present in different locations of the genome. 4-Hydroxybutyrate is most probably metabolized by a CoA transferase and a bifunctional 4-hydroxybutyryl-CoA dehydratase/vinylacetyl-CoA Δ^3^-Δ^2^-isomerase. Such enzymes and genes (*abfT* and *abfD*) have been purified and identified from *Clostridium aminobutyricum* and *Clostridium kluyveri* (11–15). Homologs of *abfD* and *abfT* are present in *C. homopropionicum*. Genes homologous to those encoding the key enzymes of the acrylyl-CoA pathway in *C. propionicum* (propionate CoA transferase [Pct] and lactoyl-CoA dehydratase [Lcd]) (16) were identified. *C. homopropionicum* can fix molecular nitrogen (1). The respective *nif* genes were found to be organized in a cluster, remarkably similar to *C. acetobutylicum* ATCC 824.

### Nucleotide sequence accession numbers.

This whole-genome shotgun project has been deposited at DDBJ/EMBL/GenBank under the accession no. LHUR00000000. The version described in this paper is version LHUR01000000.
